# Ecological Diversity, Metabolic Versatility, and Biotechnological Applications of *Burkholderia* Species: An Overview

**DOI:** 10.3390/antibiotics15010017

**Published:** 2025-12-22

**Authors:** Ali Diyapoglu, Alican Abay, Menghsiao Meng

**Affiliations:** 1 Graduate Institute of Biotechnology, National Chung Hsing University, Taichung 40227, Taiwan; diyapoglu0001@as.edu.tw (A.D.); d110041004@mail.nchu.edu.tw (A.A.); 2 Molecular and Biological Agricultural Sciences, Taiwan International Graduate Program, Academia Sinica and National Chung Hsing University, Taipei 11529, Taiwan; 3 Agricultural Biotechnology Research Center, Academia Sinica, Taipei 11529, Taiwan

**Keywords:** *Burkholderia*, *Paraburkholderia*, secondary metabolites, natural products, volatile organic compounds, drug discovery

## Abstract

*Burkholderia* is a metabolically versatile genus of Gram-negative bacteria that inhabits niches ranging from soil and water to plants and clinical environments. This review provides an integrated examination of *Burkholderia* species, focusing on their dual roles as both pathogens and beneficial microorganisms. Key pathogenic species, such as members of the *Burkholderia cepacia* complex and the *Burkholderia pseudomallei* group, pose significant threats to human, animal, and plant health due to their intrinsic antibiotic resistance and diverse virulence factors. Conversely, several environmental and plant-associated *Burkholderia* species promote plant growth, enhance nutrient uptake, and serve as biocontrol agents, supporting sustainable agriculture. We synthesize current knowledge across taxonomy, genomics, pathogenicity, beneficial interactions, and secondary metabolite biosynthesis—including the prolific production of antibiotics, toxins, and volatile organic compounds with pharmaceutical and agricultural potential. Advances in high-throughput genomics are revealing substantial genetic diversity, genome plasticity, and mechanisms underlying both pathogenicity and beneficial traits. Clarifying this dual nature and identifying strategies to mitigate risks will guide the safe and effective exploitation of *Burkholderia* in medicine, agriculture, and biotechnology.

## 1. Introduction

The genus *Burkholderia* comprises a diverse group of Gram-negative bacteria, characterized by their metabolic versatility and ability to colonize a wide range of ecological niches, including soil, water, and host-associated environments [1,2]. Notably, the genus includes both pathogenic species, which pose significant health risks, and beneficial species, which contribute to sustainable agriculture and biotechnology [3,4]. This duality has made *Burkholderia* a subject of considerable interest in medical, agricultural, and biotechnological research.

Several *Burkholderia* species are recognized as significant human, animal, and plant pathogens. Notably, members of the *Burkholderia cepacia complex* (Bcc) are major opportunistic pathogens in individuals with cystic fibrosis (CF) and other immunocompromised conditions, often leading to chronic lung infections [5,6]. *Burkholderia pseudomallei* and *Burkholderia mallei*, the causative agents of melioidosis and glanders, respectively, pose serious public health and veterinary concerns due to their high virulence and intrinsic antibiotic resistance [7,8,9]. Furthermore, certain species, such as *Burkholderia gladioli*, cause diseases in crops and contribute to significant agricultural losses [10]. Comparative genomic and functional studies have shown that pathogenic *Burkholderia* sensu stricto lineages typically harbor suites of virulence determinants—such as specialized secretion systems, capsular polysaccharides, and intracellular survival factors—that enable host invasion, immune evasion, and tissue damage [11,12,13].

In contrast, some other *Burkholderia* species contribute positively to ecosystem functioning and sustainable agriculture. Many environmental and plant-associated members of *Burkholderia* sensu lato, including strains now reclassified as *Paraburkholderia* and *Caballeronia*, generally do not harbor the virulence gene repertoires characteristic of clinical pathogens and instead encode traits linked to rhizosphere competence, nutrient acquisition, and symbiosis, such as nitrogen fixation and modulation of plant immunity [14,15,16,17]. *Burkholderia phytofirmans* (reclassified as *Paraburkholderia phytofirmans*) promotes plant growth by aiding nutrient uptake and inducing systemic resistance against pathogens [18]. Additionally, certain species can fix nitrogen, thereby improving soil fertility and supporting sustainable farming practices [19]. At the cellular level, pathogenic *Burkholderia* often invade and persist within host cells and/or cause extensive tissue damage, as shown for *B. pseudomallei* and members of Bcc in experimental and clinical studies [3,12,13,20]. In contrast, beneficial endophytes such as *P. phytofirmans* PsJN colonize root and shoot tissues without visible disease symptoms and can prime induced systemic resistance in their plant hosts [18,21].

The genus also produces a wide range of bioactive secondary metabolites, including siderophores, antibiotics, toxins, and volatile organic compounds (VOCs), which are important for drug discovery, crop protection, and ecological interactions. Some species have been studied as biocontrol agents because of their ability to suppress plant pathogens through antagonistic interactions or the production of secondary metabolites [14,15]. The genomic complexity and metabolic diversity of *Burkholderia* underpin both their adaptability and their capacity for innovation in metabolite production, environmental resilience, and symbiosis. However, the presence of multidrug resistance, the capacity for horizontal gene transfer, and the potential for pathogenicity highlight the need for careful assessment and management in both clinical and environmental contexts. Recent advances in comparative genomics, genome editing, and regulatory-network engineering are beginning to reveal and harness the genus’s hidden metabolic capacity.

Given the remarkable versatility of *Burkholderia*, as both a threat and a resource, a comprehensive understanding of its taxonomy, genomic features, pathogenic mechanisms, beneficial traits, and application potential is essential. Our literature survey primarily focused on publications from approximately 2000 to early 2025, while also incorporating earlier foundational studies that are essential for context. This review provides an integrated analysis of these aspects. By bridging knowledge from medical, agricultural, and biotechnological perspectives, we aim to clarify the risks and opportunities presented by *Burkholderia* and to guide future research toward safe and effective utilization of this complex genus.

## 2. Ecological Diversity of *Burkholderia* Species

*Burkholderia* is a genus in the Burkholderiaceae family within the order Burkholderiales, class Betaproteobacteria, and phylum Proteobacteria [16]. This genus was proposed by Yabuuchi et al. in 1992 after separation from *Pseudomonas* based on molecular and phenotypic characteristics [22]. Since then, *Burkholderia* has undergone taxonomic revisions, resulting in the reclassification of certain species into new genera such as *Paraburkholderia*, *Caballeronia*, *Robbsia*, *Mycetohabitans*, and *Trinickia* based on phylogenetic and functional distinctions [17,23,24].

**Figure 1:**
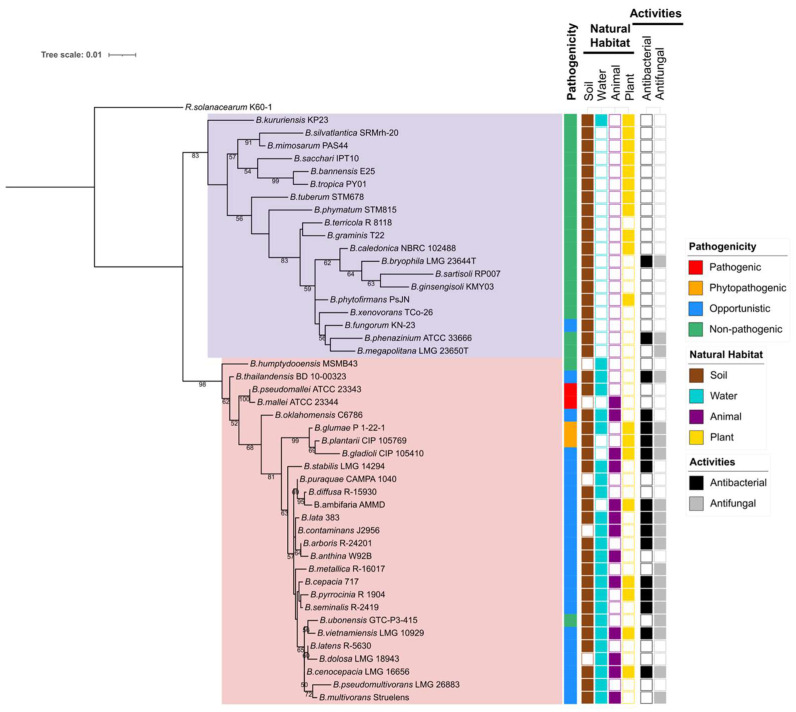
Maximum-likelihood phylogenetic tree based on 16S rRNA gene sequences of *Burkholderia* species, with *Ralstonia solanacearum* included as an outgroup. Sequence alignment and phylogenetic reconstruction were performed in MEGA 12 [25] using the Tamura–Nei evolutionary model. The resulting tree was visualized and annotated using iTOL (https://itol.embl.de/ (accessed on 13 October 2025)). Displayed features, including pathogenicity, natural habitat, and biological activities, are species-specific rather than strain-specific. White boxes in the activity column indicate that information is unavailable. Bootstrap support values (>50%) are shown at the corresponding branch nodes. Background shading: lavender/purple represents *Paraburkholderia*; pink/rose represents *Burkholderia*.

Currently, the *Burkholderia* sensu lato comprises over 100 recognized species, broadly divided into two major groups based on their ecological roles and pathogenicity: (1) Pathogenic *Burkholderia* species, such as Bcc, *B. pseudomallei*, and *B. mallei* [11,20] and (2) environmental or plant-beneficial species such as *P. phytofirmans* and *Burkholderia tropica* (reclassified as *Paraburkholderia tropica*) [18]. The *Burkholderia* species included in this review are summarized in Figure 1.

## 3. Genomic Features of *Burkholderia* Species

Genomes of *Burkholderia* species are highly complex and diverse, which explains their metabolic versatility and ability to adapt to various environments. Typically, these bacteria possess multiple chromosomes (commonly two or three) with genome sizes varying from 6 to 9 megabase pairs [26,27]. This multipartite genome structure contributes to their genetic plasticity and adaptability [28]. The GC content of *Burkholderia* genomes is relatively high (≈65–68%), and elevated GC in prokaryotes has been associated with DNA double-strand break repair-linked processes [29,30,31].

*Burkholderia* species are known for extensive horizontal gene transfer, which contributes to their antibiotic resistance, virulence, and metabolic diversity [32,33]. Pathogenic species harbor multiple virulence factors, including type III secretion system (T3SS) and type VI secretion system (T6SS), extracellular polysaccharide biosynthesis, quorum sensing (QS), and biofilm formation genes, all contributing to host infection, immune evasion, persistence, and antibiotic resistance [12,13,34,35]. Furthermore, many *Burkholderia* species have gene clusters encoding non-ribosomal peptide synthetases (NRPS) and polyketide synthases (PKS), which are responsible for producing bioactive compounds with antimicrobial properties, making them valuable for drug discovery [36]. *Burkholderia* genomes contain numerous genes associated with stress response, efflux pumps, and degradation of complex organic compounds, enabling them to survive in diverse environments, including contaminated soils and host tissues [37,38,39].

Comparative genomic studies have highlighted significant genetic diversity within *Burkholderia* sensu lato, with distinct evolutionary trajectories for pathogenic and environmental lineages. For example, the genomes of host-adapted pathogens such as *B. pseudomallei* and *B. mallei* show signs of genome reduction, pseudogene accumulation, and loss of certain environmental functions consistent with long-term adaptation to animal hosts [26,40], whereas environmental species like *Burkholderia xenovorans* (reclassified as *Paraburkholderia xenovorans*) retain large, multireplicon genomes with extensive catabolic pathways for degrading xenobiotic compounds and supporting broad metabolic versatility [37]. These contrasting patterns illustrate divergent genome-size and content trajectories for pathogenic versus environmental members of *Burkholderia* sensu lato.

Whole-genome sequencing and phylogenomic analyses have also clarified the evolutionary split between pathogenic *Burkholderia* and environmental *Paraburkholderia* lineages. In these analyses, clinically important and phytopathogenic *Burkholderia* sensu stricto (including the Bcc and the *B. pseudomallei*/*B. mallei* group) form a clade distinct from predominantly non-pathogenic, plant-associated species that have been reclassified into *Paraburkholderia* and related genera [17,23]. This phylogenetic division parallels the genomic trends described above: host-restricted pathogens generally possess more compact, reduced genomes, whereas *Paraburkholderia* species typically maintain larger genomes enriched in genes for plant association, nitrogen fixation, and other symbiotic or rhizosphere functions [17,23].

## 4. Pathogenicity of *Burkholderia* Species

Several *Burkholderia* species are significant pathogens, causing severe infections in humans, animals, and plants. The pathogenicity of *Burkholderia* is linked to its ability to survive in diverse environments, evade host immune responses, and resist antibiotics. At the genetic level, these pathogenic lineages carry characteristic virulence loci, including T3SS and T6SS and high-affinity siderophore biosynthesis with transport genes. Examples include T3SS-1 and T6SS-1 in *B. pseudomallei* and the ornibactin biosynthesis and transport genes in Bcc species; collectively, these systems promote intracellular survival, immune evasion, and systemic infection [12,13,41,42]. The primary pathogenic species can be grouped as follows:

### 4.1. Human and Animal Pathogens

The Bcc consists of at least 20 closely related species, including *Burkholderia cepacia*, *Burkholderia cenocepacia*, and *Burkholderia multivorans*, which are opportunistic pathogens primarily affecting individuals with CF and other immunocompromised conditions [11,43,44]. Some infections progress to cepacia syndrome, an acute, rapidly progressive necrotizing pneumonia characterized by high fever and bacteremia and associated with high mortality [45,46,47].

*B. pseudomallei*, commonly found in soil and water, is the causative agent of melioidosis, a severe infectious disease endemic to Southeast Asia and northern Australia [20]. Melioidosis presents with a range of symptoms, including pneumonia, abscess formation, sepsis, and neurological complications, and can be fatal; mortality is ~10% in settings with rapid diagnosis, optimal therapy, and intensive care, but is ≥40% in many endemic regions where such resources are limited [20,48].

*B. mallei* is a host-adapted pathogen responsible for glanders, primarily affecting horses, donkeys, and mules, though humans can also be infected. In equines, glanders is characterized by ulcerating nodular lesions of the skin and mucous membranes with generalized signs such as fever and cough [49]. In humans, glanders presents as pulmonary, cutaneous, or septicemic infections, with a high fatality rate if untreated. *B. mallei* has been classified as a biodefense concern due to its prior use as a biological weapon and its high case-fatality rate [50].

### 4.2. Plant Pathogens

*B. gladioli* is a plant-associated bacterium with a broad host range, including rice, orchidaceous plants, gladiolus, onions, and mushrooms [51,52]. The species comprises multiple pathovars (pv.); pv. *gladioli*, pv. *alliicola*, pv. *agaricicola*, and pv. *cocovenenans*. Pathovars pv. *gladioli*, pv. *alliicola*, and pv. *agaricicola* are linked to soft-rot diseases of gladiolus, onion bulbs, and mushrooms, respectively, with pv. *agaricicola* recognized as an important mushroom pathogen due to its potential to cause major crop losses. In contrast, pv. *cocovenenans* is distinguished by its association with toxic food spoilage [52]. In rice, *B. gladioli* is associated with bacterial grain rot and leaf-sheath browning. It has also been isolated from healthy rice plants and rice nursery soil, supporting its ability to persist in agricultural environments [51].

*Burkholderia glumae* is an important phytopathogen of rice that causes bacterial panicle blight and seedling rot, leading to significant economic losses [53,54,55]. Disease symptoms commonly include grain discoloration, spikelet sterility (failure of grain filling), and seedling rot, which severely reduce crop yields [53,55].

### 4.3. Treatment and Control

Treatment and control of infections caused by *Burkholderia* species pose significant clinical and agricultural challenges due to their inherent multidrug resistance and diverse pathogenic strategies. For infections caused by the Bcc, therapy is typically individualized and combination-based according to in vitro susceptibility testing. Commonly used agents include ceftazidime, trimethoprim-sulfamethoxazole (TMP-SMX), meropenem or levofloxacin [56], but in vitro resistance of Bcc species to these drugs is frequent, underscoring the need for susceptibility-guided regimens [57,58]. Emerging approaches to address resistance include the use of ceftazidime-avibactam [59] and, in highly resistant cases, a combination of ceftazidime-avibactam, ciprofloxacin, meropenem, minocycline, sulfadiazine, and tobramycin [60]. Bacteriophage therapy is under active investigation as an adjunct or alternative approach [61]. Strict infection control measures remain essential, especially for CF patients and immunocompromised individuals.

Management of melioidosis involves intensive intravenous antibiotic therapy (e.g., ceftazidime, meropenem, or imipenem) for at least 2 weeks, depending on severity, followed by oral eradication therapy (3 to >6 months) with TMP-SMX as a first-line; doxycycline or amoxicillin/clavulanic acid as alternatives [20,48]. Public health measures such as reducing exposure to contaminated soil and water, wearing protective clothing, and raising public awareness are essential in endemic regions.

For glanders, antibiotic therapy typically includes doxycycline, ceftazidime, or TMP-SMX [49,50]. Control relies heavily on stringent biosecurity measures, including early detection, quarantine, and culling of infected animals to prevent transmission [49,50]. Given its potential as a bioterrorism agent, robust surveillance and adherence to biosecurity protocols are vital [50].

Control of *B. gladioli*–induced plant diseases relies primarily on integrated cultural practices: plant only scab-free corms, avoid replanting in previously affected soils, implement multi-year rotations (≈3 years), improve drainage and sanitation, and manage bulb mites that create infection courts [62,63]. Because copper and streptomycin resistance is common in *B. gladioli*, bactericides have limited and inconsistent efficacy and should not be relied on as primary control measures [64]. As a biological option, prophylactic bacteriophage treatments—for example, *Burkholderia* phages KS12 and AH2—have been shown to prevent or reduce *B. gladioli*–associated tissue destruction in a quantitative ex planta model, offering a promising eco-friendly adjunct to cultural controls [65].

Disruption of the bacterial proton motive force—by genetic manipulation or by chemical means such as sodium bicarbonate—significantly reduces the virulence of *B. glumae* in rice [66]. Additionally, biological control strategies show promise: seed/seedling treatments using *Cytobacillus firmus* JBRS159 in combination with silicon (SiO_2_ nanoparticles or K_2_SiO_3_) suppressed bacterial seedling rot of rice plants [67]. A flagella-dependent jumbo phage (S13) requires *B. glumae* flagella for attachment and infection, lysing flagellated cells and selecting for nonflagellated survivors with reduced virulence—an effect that protects rice seedlings [68]. These advances provide practical solutions towards integrated and sustainable management of *B. glumae*-associated rice diseases.

## 5. Environmental and Beneficial *Burkholderia* Species

In contrast to pathogenic members of *Burkholderia* sensu stricto, some members of *Burkholderia* sensu lato (including *Paraburkholderia*) play beneficial roles in agriculture and environmental sustainability. Notably, *P. phytofirmans* and *P. tropica* have been recognized for their plant growth-promoting and biocontrol properties.

*P. phytofirmans*, particularly the strain PsJN, is a well-studied endophytic bacterium capable of colonizing diverse plant hosts (e.g., grapevine, tomato, and potato). It promotes plant growth through traits including phytohormone-associated pathways (notably indole-3-acetic acid), 1-aminocyclopropane-1-carboxylate deaminase activity, and siderophore production. PsJN can also enhance protection against phytopathogens by inducing plant-mediated systemic resistance and improve tolerance to abiotic stresses such as drought and cold [21].

*P. tropica* is a plant-associated diazotrophic bacterium isolated from the rhizosphere and endophytic compartments of crops, including maize, sugarcane, and teosinte. This species exhibits nitrogenase activity under microaerobic conditions in nitrogen-free media, supporting its capacity for biological nitrogen fixation [69].

These beneficial functions are supported by characteristic gene modules in plant-associated *Paraburkholderia*. Diazotrophic taxa such as *P. tropica* and the legume symbiont *Paraburkholderia phymatum* harbor conserved nitrogen-fixation genes (e.g., *nifHDK* and associated *nif* genes), while *P. phymatum* also carries *nod* gene sets (e.g., *nodABCD*) linked to nodulation [15,17]. Together, these genetic modules underpin rhizosphere/endophytic competence and biological nitrogen fixation that can enhance plant nutrient status and stress resilience [15,17,21].

## 6. Secondary Metabolites Produced by *Burkholderia* Species

*Burkholderia* species are prolific producers of diverse secondary metabolites, which have significant implications for both ecology and medicine. These metabolites contribute to the genus’s ability to thrive in diverse environments, suppress competitors, interact with plant hosts, and, in some cases, cause disease. An overview of secondary metabolites discussed in this review and their producing species is summarized in Table 1.

### 6.1. Siderophores

Siderophores produced by *Burkholderia* species are critical for iron acquisition, especially under iron-limiting conditions encountered in natural and host-associated environments [70]. These compounds not only support microbial survival and competitiveness but also contribute to pathogenicity and ecological adaptability.

#### 6.1.1. Cepaciachelin

Cepaciachelin is notably produced by *B. cepacia* and *B. ambifaria*. By sequestering iron from the environment, cepaciachelin enables producing strains to thrive in niches where iron availability is limited, thereby contributing to their ecological success and pathogenic potential [70,71,128]. In clinical settings, particularly among immunocompromised individuals or those with CF, such mechanisms may contribute to the persistence and virulence of these bacteria [32].

#### 6.1.2. Ornibactin

Ornibactin is produced by various *Burkholderia* species, notably *B. cenocepacia* and *B. contaminans*. These bacteria utilize ornibactin to acquire iron from the iron-limited environment, such as that within a host organism [41,72,73]. Mutants deficient in ornibactin production exhibit impaired growth under iron-limited conditions and reduced virulence in animal models. For instance, *B. cepacia* mutants lacking ornibactin biosynthesis were less effective in establishing infections in murine respiratory models [73].

Ornibactin is a linear hydroxamate-hydroxycarboxylate siderophore, composed of a tetrapeptide backbone: _L_-Orn^1^(*N*^δ^-OH, *N*^δ^-acyl)-_D_-*threo*-Asp(β-OH)-_L_-Ser-_L_-Orn^4^(*N*^δ^-OH, *N*^δ^-formyl)-1,4-diaminobutane. The terminal ornithine residues are modified with hydroxyl and acyl groups, with the acyl chains varying in length (e.g., 3-hydroxybutanoic acid, 3-hydroxyhexanoic acid, and 3-hydroxyoctanoic acid), leading to different ornibactin variants such as ornibactin-C_4_, ornibactin-C_6_, and ornibactin-C_8_ [73].

The biosynthesis of ornibactin is mediated by NRPSs, specifically OrbI and OrbJ. These enzymes sequentially assemble the ornibactin molecule by incorporating and modifying amino acid substrates. The process is regulated by the extracytoplasmic function sigma factor OrbS, which controls the expression of ornibactin biosynthesis and transport genes in response to iron availability [42].

Recent studies have explored the potential of ornibactin in diagnostic imaging. A radiolabeled form of ornibactin complexed with gallium-68, [^68^Ga]Ga-ornibactin, has been developed for positron emission tomography imaging to detect *Burkholderia* infections. This approach demonstrated specific accumulation of the radiotracer at infection sites in animal models, suggesting its utility in diagnosing and monitoring infections caused by the Bcc [129].

#### 6.1.3. Malleobactin

Malleobactin is notably produced by *B. pseudomallei*, *B. mallei*, and *B. thailandensis* [28,75]. The biosynthesis of malleobactin is directed by the *mba* gene cluster, which encodes NRPSs responsible for assembling the siderophore. Notably, the NRPS involved in malleobactin production exhibits a rare flexibility, yielding diverse peptide backbones and resulting in various malleobactin congeners [75]. Malleobactin is characterized by its unusual peptide backbone that includes the rare 2-amino-5-nitropentanoic acid, which was reported for the first time as a natural product building block in this siderophore [76].

In pathogenic *Burkholderia* species, malleobactin plays a crucial role in iron acquisition, facilitating survival and proliferation within iron-restricted environments like the human host. However, studies have shown that while malleobactin contributes to iron uptake, it is not solely responsible for virulence. For instance, *B. pseudomallei* strains deficient in malleobactin production remain virulent in murine models, suggesting the presence of alternative iron acquisition systems that compensate for the loss of malleobactin [130]. 

The expression of malleobactin biosynthesis genes is tightly regulated in response to iron availability. Under iron-limiting conditions, the extracytoplasmic function sigma factor MbaS upregulates the transcription of genes involved in malleobactin production and transport. This regulatory mechanism ensures that siderophore production is induced when iron is scarce, optimizing bacterial survival strategies [74].

#### 6.1.4. Bolagladins

Bolagladins are a class of lipodepsipeptides produced by *B. gladioli* isolates BCC0238 and BCC1622 from the lungs of CF patients. These metabolites exhibit distinctive structural features, including a citrate-derived fatty acid and a rare dehydro-β-alanine residue [77]. The biosynthesis of bolagladins is orchestrated by a cryptic NRPS gene cluster. Through a combination of bioinformatics analyses and genetic experiments, researchers elucidated the mechanisms responsible for incorporating the distinctive citrate-derived fatty acid and the dehydro-β-alanine residue into the bolagladin structure [77].

#### 6.1.5. Gramibactin

Gramibactin is a novel class of diazeniumdiolate (N-nitroso-N-hydroxylamine) siderophore produced by certain *Burkholderia* species, notably *P. graminis*, a bacterium associated with cereal rhizospheres [78].

Structurally, gramibactin is a lipodepsipeptide composed of six amino acids, including two D-graminine (Gra) residues, along with glycine, D-allo-threonine, L-threonine, and D-threo-β-hydroxyaspartic acid. The presence of the rare diazeniumdiolate moiety in graminine is pivotal for its iron-binding capability. Biosynthetically, graminine originates from L-arginine, with the N-N bond in the diazeniumdiolate formed between the Nδ and Nω atoms of the guanidinium group in L-arginine [131]. The diazeniumdiolate groups in gramibactin exhibit a strong affinity for ferric ions, facilitating efficient iron sequestration. This high-affinity binding is crucial for the survival of *P. graminis* in iron-limited environments, such as the rhizosphere [79]. 

Beyond iron acquisition, gramibactin’s diazeniumdiolate moiety can release nitric oxide (NO), a signaling molecule involved in various plant physiological processes. This NO release may enhance plant growth and stress tolerance, suggesting a symbiotic relationship between *P. graminis* and its host plants [80]. The dual functionality of gramibactin, as an iron chelator and NO donor, opens avenues for its application in agriculture, particularly as a biofertilizer to improve plant health and yield. Additionally, understanding its biosynthesis could lead to the development of novel compounds with therapeutic or industrial relevance.

### 6.2. Antibiotics

*Burkholderia* species produce a wide array of antimicrobial substances as a competitive strategy to survive and thrive in complex microbial communities. At subinhibitory concentrations, some antibiotics may act as signaling molecules affecting the inter- and intraspecies communication. Many antibiotics produced by *Burkholderia* species exhibit antagonistic activity against drug-resistant nosocomial bacterial pathogens through novel modes of action, highlighting their potential use in the medical sector. However, additional preclinical and clinical studies are needed to assess their efficacy and safety.

#### 6.2.1. Gladiolin

Gladiolin is a macrolide antibiotic initially discovered from *B. gladioli* BCC0238 through genome mining and metabolomic approaches. Its structure features a large cyclic lactone ring with a complex pattern of unsaturated and oxygenated functionalities [81].

Gladiolin biosynthesis occurs via modular type-I PKS pathways. This biosynthetic gene cluster (BGC) encodes a *trans*-AT PKS system; an atypical feature compared with the more common *cis*-AT systems found in other bacterial macrolide producers [81,132]. Gladiolin exhibits potent activity against Gram-positive bacteria, notably drug-resistant *Mycobacterium tuberculosis*. Gladiolin acts by selectively targeting bacterial RNA polymerase, disrupting transcription, thereby inhibiting bacterial growth [81]. This mode of action differs from many conventional antibiotics, offering promise for overcoming resistance mechanisms prevalent among multidrug-resistant pathogens.

#### 6.2.2. Enacyloxin

Enacyloxins are polyketide antibiotics, exhibiting significant antimicrobial activity. They are produced by certain *Burkholderia* species, notably *B. ambifaria*, through an unusual hybrid modular PKS pathway, highlighting the bacterium’s capacity to produce diverse bioactive compounds [83]. Among its congeners, enacyloxin IIa is notable for its potent activity against multidrug-resistant pathogens and represents a promising candidate for new antimicrobial development [82,83].

#### 6.2.3. Gladiostatin

Gladiostatin, also known as gladiofungin, was initially discovered in *B. gladioli* strains BCC0238 and BCC1622. It is a glutarimide-containing polyketide with a terminal 2-acyl-4-hydroxy-3-methylbutenolide moiety, a structural feature that differentiates gladiostatin from the other glutarimide antibiotics previously described from *Streptomyces* species [85,133]. The biosynthesis of gladiostatin is orchestrated by a *trans*-AT PKS pathway. Notably, an AfsA-like domain at the C-terminus of the PKS plays a crucial role by catalyzing the condensation of 3-ketothioesters with dihydroxyacetone phosphate, providing a noncanonical polyketide chain-release mechanism that installs the butanolide side chain [85,86]. This compound displays both antifungal and antiproliferative activity against human cancer cell lines [85,86].

#### 6.2.4. Icosalide

Icosalide is a distinctive lipopeptidiolide antibiotic produced by certain *Burkholderia* species. Originally, icosalide A1 was isolated from the fungus *Aureobasidium* sp. MSX 59166. However, further investigations uncovered that the true producers were *B. gladioli* strains associated with the fungal cultures. This finding highlighted the intricate relationships between fungi and their bacterial symbionts [87,88]. Icosalide A1 is characterized by a 20-membered lipopeptidiolide ring, comprising two serine and two leucine residues, along with 3-hydroxy fatty acid chains of varying lengths. The presence of a D-leucine residue in icosalide A1 distinguishes it from its analogs, icosalide A2 and icosalide B, which contain only L-amino acids [87].

The biosynthesis of icosalide is orchestrated by an NRPS encoded by the *icoA* gene. This NRPS exhibits an unusual architecture, featuring two chain-initiating condensation (C_I) domains. One C_I domain is located at the N-terminus of module 1, a common feature in lipopeptide assembly, while the other is embedded within module 3. This architecture enables the initiation of two separate lipopeptide chains, which are subsequently joined to form the asymmetric diolide structure of icosalide [88].

Icosalide A1 exhibits antimicrobial properties, particularly against Gram-positive bacteria such as *Streptococcus pyogenes*. The incorporation of a D-leucine residue is crucial for its antibacterial activity, as analogs lacking this residue do not display similar efficacy. Additionally, icosalide A1 has been shown to inhibit swarming motility in certain bacterial species, suggesting a role in modulating microbial behavior [87].

#### 6.2.5. Burkholdines

Burkholdines were first isolated from *B. ambifaria* strain 2.2N. This bacterium was found to produce two primary variants: burkholdine 1229 (Bk-1229) and burkholdine 1097 (Bk-1097). Both compounds demonstrated significant antifungal activity against a range of fungal pathogens, with potencies surpassing that of amphotericin B, a commonly used antifungal agent [89,134].

These NRPS-derived compounds are characterized by their cyclic octapeptide structure, incorporating nonproteinogenic amino acids such as β-hydroxytyrosine and β-hydroxyasparagine. Additionally, they contain a characteristic fatty acyl amino acid moiety that contributes to their amphipathic nature, which is crucial for their interaction with fungal cell membranes [90].

#### 6.2.6. Pyrrolnitrin

Pyrrolnitrin is a halogenated phenylpyrrole antibiotic initially isolated from *Pseudomonas pyrrocinia*. It has since been identified in several other bacterial genera, including *Burkholderia*, notably *B. cepacia*. Pyrrolnitrin is characterized by its chlorinated pyrrole structure, specifically 3-chloro-4-(3-chloro-2-nitrophenyl)-1H-pyrrole. In *Burkholderia* species, the biosynthesis of pyrrolnitrin involves the conversion of tryptophan through a series of enzymatic steps, leading to the formation of this bioactive compound [94].

Pyrrolnitrin exhibits potent antifungal activity against a wide range of filamentous fungi and yeasts. Additionally, it shows efficacy against certain Gram-positive bacteria, including *Streptomyces* species. The compound functions by inhibiting the electron transport chain in target organisms and disrupting cellular respiration, leading to cell death [91,94].

Pyrrolnitrin’s agricultural relevance is demonstrated by its biocontrol efficacy, as shown in *B. cepacia* strain 5.5B, which suppresses *Rhizoctonia solani*-induced stem rot in poinsettia [135]. In addition, *B. cepacia* strain K87 has been shown to produce novel oxidized derivatives of pyrrolnitrin, contributing to the discovery of structurally diverse antifungal agents with potentially improved activities [136].

#### 6.2.7. Cepacin

Cepacin A and B are acetylenic antibiotics, containing a series of conjugated triple bonds in their carbon backbone. Cepacin exhibits significant antifungal activity, effectively inhibiting the growth of various phytopathogenic fungi [95]. In biological assays, *B. ambifaria* strains producing cepacin were able to suppress damping-off diseases caused by *Globisporangium ultimum* (formerly *Pythium ultimum*) in peas, showcasing its effectiveness in real-world agricultural scenarios [137].

#### 6.2.8. Thailandenes

Thailandenes are a class of polyene natural products produced by *B. thailandensis*. The biosynthetic pathways responsible for thailandene production involve a series of enzymatic reactions that assemble these complex molecules from simpler precursors [96].

While the full spectrum of thailandene biological activities is still under investigation, preliminary studies suggest that these compounds may possess antimicrobial properties. Their polyene structures are known to interact with microbial membranes, potentially leading to antibacterial effects.

#### 6.2.9. Thailandamide

Thailandamide is also a polyene natural antibiotic produced by *B. thailandensis*. It inhibits fatty acid biosynthesis by targeting acetyl-CoA carboxylase (AccA), an early, essential enzyme in the pathway [98]. Consistent with this target, thailandamide shows strong antibacterial activity against Gram-positive and cell wall-weakened Gram-negative bacteria [138].

Thailandamide biosynthetic genes co-occur with a duplicate, resistant *accA* allele (*accA2*), which provides producer self-resistance [98]. This self-resistance mechanism is critical for the survival of *B. thailandensis* when producing thailandamide. The biosynthesis of thailandamide is tightly regulated by QS, with QS mutants exhibiting altered secondary-metabolite profiles [99,139]. In addition, LysR-type regulator (LTTR) ScmR broadly influences secondary metabolism in *B. thailandensis*, linking thailandamide to a larger regulatory network [140].

#### 6.2.10. Bactobolin

Bactobolin is an antibiotic produced by *B. thailandensis* whose biosynthesis is coordinated by an N-acyl homoserine lactone QS system in response to cell density, and the corresponding BGC has been well characterized in *B. thailandensis* strain E264 [100,141].

Bactobolin inhibits translation, and resistance to bactobolin can be conferred by mutations in ribosomal protein L2, supporting the ribosome as its primary target [101]. This mode of action is distinct from many classical antibiotics, highlighting bactobolin’s potential as a lead compound for antibiotic development [101].

#### 6.2.11. 4-Hydroxy-3-methyl-2-alkylquinolines (HMAQs)

HMAQs are specialized secondary metabolites produced primarily by species within the *B. pseudomallei* group, including *B. thailandensis*, *B. pseudomallei*, and closely related species [102,141]. These molecules share structural similarities with 4-hydroxy-2-alkylquinolines produced by *Pseudomonas aeruginosa*, but instead possess a methyl substitution at the 3-position of their quinoline ring, distinguishing their chemical properties and biological activities [102,141].

The biosynthetic pathway responsible for HMAQ production in *Burkholderia* involves the *hmqABCDEFG* operon, analogous to the *pqs* operon found in *P. aeruginosa*. However, the *hmq* operon is specifically adapted within *Burkholderia* species to yield methylated quinoline derivatives [102]. This operon has been well characterized in *B. thailandensis*, *B. pseudomallei*, and *B. ambifaria*, demonstrating its conserved presence among diverse members of this genus [102].

HMAQs exhibit notable antimicrobial activity, particularly against Gram-positive bacteria such as *Staphylococcus aureus*, as well as certain fungal species [142]. The antimicrobial activity of HMAQs and their N-oxide derivatives underscores their ecological function, enabling *Burkholderia* to compete effectively within soil, plant-associated environments, and potentially clinical niches by suppressing microbial competitors [141].

Apart from antimicrobial activity, HMAQs are also involved in complex QS regulatory networks within *Burkholderia* species. Specifically, studies on *B. thailandensis* strains have demonstrated that HMAQs contribute significantly to the regulation of genes associated with biofilm formation, motility, and stress responses [140,143,144], emphasizing their role in bacterial survival and environmental adaptation.

#### 6.2.12. Occidiofungin

Occidiofungin is a cyclic glycopeptide exhibiting potent antifungal activity, produced primarily by the bacterium *B. contaminans* strain MS14. It shows significant activity against a wide range of fungal pathogens, including *Candida* species and the mold *Aspergillus flavus* [145,146]. Structurally, occidiofungin consists of unusual amino acid residues and glycosylation, contributing to its distinctive biochemical properties and antifungal activity [104]. Its potent antifungal efficacy arises from a novel mechanism of action involving binding to actin, a key cytoskeletal protein that disrupts actin polymerization, ultimately leading to fungal cell death [107].

The biosynthesis of occidiofungin involves several genes within a well-characterized BGC. Genetic studies revealed that the cluster includes genes encoding NRPSs, regulatory elements, and specific enzymes such as the xylosyltransferase encoded by the *ocfC* gene, which is essential for occidiofungin’s glycosylation [105,147]. Variants of occidiofungin, designated as OCF-E through OCF-J, have been identified and characterized, expanding the known structural diversity and biological potential of this metabolite [108].

### 6.3. Toxins

#### 6.3.1. Bongkrekic Acid

Bongkrekic acid is produced by *B. gladioli* pv. *cocovenenans*, a strain historically associated with severe food poisoning outbreaks, particularly in Southeast Asia. It is a respiratory toxin that inhibits mitochondrial adenine nucleotide translocase, a key protein responsible for the exchange of ADP and ATP across the inner mitochondrial membrane. This inhibition disrupts cellular energy homeostasis, leading to rapid bioenergetic failure and potentially fatal outcomes. The compound was first identified following fatal poisonings linked to consumption of fermented coconut-based foods, notably “tempe bongkrèk”—a traditional Indonesian food. Hence, the toxin was named “bongkrekic acid” [148,149,150]. Over the past decade, bongkrekic acid poisoning has been documented in a large outbreak in Mozambique linked to a traditional African beverage, in multiple foodborne incidents in China, and, more recently, in the first confirmed cases reported in North America and Taiwan, underscoring its emergence as a global food-safety threat [150,151,152,153,154,155].

Structurally, bongkrekic acid is a highly unsaturated tricarboxylic acid. It is extremely stable, heat-resistant, and unaffected by typical cooking processes, significantly complicating control measures once contamination occurs [156]. Human exposure to bongkrekic acid typically occurs through ingestion of contaminated food products. Symptoms manifest rapidly, often within hours of ingestion, and include severe vomiting, nausea, dizziness, headaches, epigastric discomfort, jaundice, and neurological symptoms such as seizures and unconsciousness. Reported mortality rates vary widely, with values ranging from 30% to 100% depending on the outbreak [150,156]. Due to its potency and stability, bongkrekic acid poisoning remains a serious public health concern, particularly in regions where traditional fermentation practices are prevalent.

The bongkrekic acid produced by *B. gladioli* pv. *cocovenenans* is best known for its role in severe food poisoning outbreaks, while its broader ecological role remains poorly understood [110]. Detecting bongkrekic acid contamination is crucial for preventing foodborne outbreaks. Analytical methods such as HPLC and mass spectrometry have been employed successfully to identify and quantify bongkrekic acid in contaminated food samples [157].

Despite its toxicity, the potent inhibitory action of bongkrekic acid on mitochondrial function has found niche applications in biochemical research, specifically for studying mitochondrial ATP transport and energy metabolism in cellular models. It serves as an important research tool for exploring the mitochondrial membrane function and bioenergetics in both physiological and pathological contexts [158].

#### 6.3.2. Toxoflavin

Toxoflavin acts as a phytotoxin, contributing to plant diseases such as rice grain rot and wilt in various crops [111]. Toxoflavin is a potent secondary metabolite originally isolated in 1934 from the bacterium *B. gladioli*, initially identified as *Pseudomonas cocovenenans*. It is a yellow-colored azapteridine compound recognized primarily for its phytotoxicity, contributing to severe agricultural diseases and posing challenges in crop production, especially rice cultivation [112,159]. *B. gladioli*, along with other related species, synthesizes toxoflavin as a significant virulence factor to infect host plants and compete within ecological niches [159].

The biosynthesis of toxoflavin is mediated by a dedicated gene cluster identified in the genomes of toxin-producing *Burkholderia* species, particularly *B. gladioli* and *B. glumae*. This cluster encodes several enzymes involved in a pathway starting from glycine and guanosine triphosphate. The biosynthetic mechanism involves multiple enzymatic steps, including cyclization, methylation, and oxidation reactions, ultimately yielding toxoflavin. Key enzymes characterized in this pathway include methyltransferases and oxidases, which together shape the chemical structure of the final compound [160,161].

Toxoflavin primarily exerts its biological effects through oxidative stress, mediated by the generation of reactive oxygen species, such as hydrogen peroxide and superoxide radicals. Its redox-active nature enables toxoflavin to participate in redox cycling reactions, disrupting cellular redox homeostasis. In plants, toxoflavin causes extensive damage to tissues, leading to symptoms such as necrosis, chlorosis, and wilting. The compound’s potent phytotoxicity is prominently involved in diseases such as rice grain rot and bacterial wilt in numerous crops [111,114]. This significant phytotoxicity poses a considerable threat to agriculture, particularly in rice-producing regions. Effective management strategies include developing disease-resistant crop varieties, employing good agricultural practices, seed sterilization, soil fumigation, and biological control strategies utilizing antagonistic microorganisms to suppress pathogen populations and detoxify soils contaminated with toxoflavin [115,162].

Beyond its role as a phytotoxin, toxoflavin has also been explored as a bioactive scaffold for therapeutic and agrochemical development, including anticancer, antimicrobial, and herbicidal leads. Genome mining and synthetic biology approaches are being used to re-engineer toxoflavin biosynthesis to generate derivatives with altered activity and reduced toxicity, paving the way for potential biotechnological applications [162].

#### 6.3.3. Malleilactone

Malleilactone is a polyketide-derived secondary metabolite produced by species within the *B. pseudomallei* group, including *B. pseudomallei* and *B. thailandensis*. This compound is recognized as a key virulence factor, contributing to the pathogenicity of *B. pseudomallei*, the causative agent of melioidosis [116,117]. The biosynthesis of malleilactone is directed by a PKS gene cluster, which is conserved among *B. pseudomallei* group pathogens [116].

Malleilactone production is tightly regulated by QS systems and can also be induced by exposure to certain antibiotics [117,163]. Specifically, the orphan LuxR-family regulator MalR in *B. thailandensis* can activate malleilactone biosynthesis independently of acyl-homoserine lactones, demonstrating the complex regulation of this cytotoxin [163]. These findings highlight the importance of malleilactone both in microbial competition and in the ability of *B. pseudomallei* group bacteria to cause disease.

#### 6.3.4. Thailanstatin

Thailanstatins are a group of natural products produced by *B. thailandensis* MSMB43. They exhibit potent antiproliferative activities through inhibition of pre-mRNA splicing. These compounds are structurally related to FR901464, a known splicing inhibitor, but they possess enhanced stability, making them promising candidates for anticancer drug development [118]. Thailanstatins are characterized by a hybrid polyketide-nonribosomal peptide structure. Compared with FR901464, they lack the C1 hydroxyl group associated with the hemiketal center and instead possess an additional carboxyl moiety at C17; together these differences render thailanstatins significantly more stable than FR901464 under physiologically relevant conditions [118,119]. 

Thailanstatins bind to the SF3b subcomplex of the U2 small nuclear ribonucleoprotein within the spliceosome. In vitro assays demonstrated that thailanstatins inhibit pre-mRNA splicing with half-maximal inhibitory concentrations in the single to sub-micromolar range. Cell culture assays indicated potent antiproliferative activities against various human cancer cell lines, with half-maximal growth inhibitory concentrations in the single nanomolar range [118]. Efforts have been made to improve the production yields of thailanstatins through metabolic engineering of the biosynthetic pathway in *B. thailandensis*, facilitating further preclinical studies [164,165].

#### 6.3.5. Rhizoxin

Rhizoxin is a potent antimitotic macrolide initially attributed to the fungus *Rhizopus microsporus*, the causative agent of rice seedling blight. However, subsequent research unveiled that the true producers of rhizoxin are endosymbiotic bacteria residing within the fungal cytosol, specifically *B. rhizoxinica* (reclassified as *Mycetohabitans rhizoxinica*) [17,166]. The symbiosis between *B. rhizoxinica* and *Rhizopus microsporus* is integral to the biology and virulence of the fungal host, because the bacterium supplies rhizoxin, which *R. microsporus* uses as a virulence factor in rice seedling blight. Additionally, *B. rhizoxinica* is essential for the fungal host’s vegetative spore formation, highlighting a mutualistic relationship where the bacterium provides chemical weaponry while the fungus provides protection [122,167,168].

Structurally, rhizoxin contains a 16-membered macrolactone ring linked to an oxazole moiety via a long unsaturated aliphatic chain. This structure enables rhizoxin to bind β-tubulin, resulting in inhibition of microtubule assembly and cell cycle arrest [169]. The BGC responsible for rhizoxin production encodes a hybrid PKS and NRPS assembly line, facilitating the incorporation of various building blocks into the rhizoxin molecule [120,122].

Because of its antimitotic properties, rhizoxin has been investigated as a potential anticancer agent. Phase II clinical trials in patients with advanced breast cancer and melanoma demonstrated that rhizoxin could be safely administered intravenously at a dose of 2.0 mg/m^2^ every 3 weeks. However, its therapeutic efficacy was limited, with minimal antitumor activity observed in both patient groups. This outcome, along with issues related to drug stability and toxicity, has hindered its further clinical development [121].

### 6.4. Other Metabolites

#### Rhamnolipids

Rhamnolipids are glycolipid biosurfactants produced by several *Burkholderia* species, with structures and properties that make them valuable for various industrial and environmental applications. The genetic regulation of rhamnolipid biosynthesis in *Burkholderia* involves *rhl* gene clusters, most notably *rhlA*, *rhlB*, and *rhlC*. In *B. thailandensis*, two identical *rhl* gene clusters have been identified, and these clusters encode enzymes responsible for rhamnolipid biosynthesis. The regulation of these genes is closely linked to QS systems and environmental conditions, indicating a tightly controlled genetic mechanism that responds to both cell density and external factors [127]. In *B. glumae*, the rhamnolipid biosynthetic pathway is also under the control of *rhlA*, *rhlB*, and *rhlC* homologs, and the production profile can differ from that of other species, reflecting differences in genetic regulation and metabolic potential [125].

The purity and composition of rhamnolipid preparations can be influenced by the cultivation medium, carbon source, and process conditions, which have been optimized in studies with *B. glumae* and *Burkholderia kururiensis* to maximize yield and tailor the congener profile to specific applications [125,170,171]. *Burkholderia plantarii* DSM 9509^T^ was found to produce rhamnolipids under various culture conditions, with yields and congener patterns distinct from those of *Pseudomonas* species. The study highlighted that although the total yield may be lower, the specific structural forms of rhamnolipids produced by *B. plantarii* can have characteristic physicochemical properties that are of interest for specialized applications [172].

The ongoing interest in green technologies has further increased the demand for microbial biosurfactants, including those from *Burkholderia*, for environmentally friendly applications [173]. Nevertheless, safety assessments are crucial since some *Burkholderia* species are considered opportunistic pathogens, and the use of wild-type or genetically modified strains in large-scale production requires careful risk evaluation.

## 7. Volatile Organic Compounds (VOCs) Produced by *Burkholderia* Species

VOCs are small molecules characterized by high vapor pressure and low boiling points, enabling them to evaporate quickly and diffuse readily through air and soil [174]. *Burkholderia* species synthesize a broad spectrum of VOCs with important ecological, biotechnological, and agricultural functions. These VOCs include sulfur-containing compounds, alcohols, ketones, pyrazines, hydrocarbons, and aromatic compounds, which play roles in microbial interactions, plant growth, pathogen inhibition, and communication. A summary of VOCs is provided in Table 2; and selected examples are detailed below.

### 7.1. Dimethyl Disulfide (DMDS)

Dimethyl disulfide (DMDS), characterized by a strong garlic-like odor, exhibits potent antimicrobial, antifungal, and nematicidal properties, making it a promising agent in agriculture for biocontrol purposes. DMDS production has been prominently observed in *B. gladioli*, *B. ambifaria*, and various strains within the Bcc [175,176,182].

DMDS production in *Burkholderia* species is typically associated with the catabolism of sulfur-containing amino acids, particularly methionine and cysteine. Enzymatic processes involving methionine-γ-lyase or cysteine desulfhydrase result in volatile sulfur-containing by-products, with DMDS as a prominent end product. This metabolic pathway allows the bacteria to detoxify excess sulfur and regulate intracellular sulfur pools [182,187].

*B. ambifaria* H8 was shown to inhibit *Fusarium graminearum* and protect maize plants against stalk rot through DMDS production [175]. *B. gladioli* strain BBB-01 has demonstrated fungicidal activity through DMDS and other VOCs [176]. DMDS also exhibits significant toxicity toward root-knot nematode *Meloidogyne incognita* [183,184]. Beyond antifungal and nematicidal properties, DMDS has demonstrated antibacterial and insecticidal activities. Its sulfurous nature disrupts microbial metabolism, inhibits pathogenic bacterial growth, and deters insect herbivory [188].

Given its broad-spectrum biological activities, DMDS offers a potential alternative to conventional chemical fumigants. In fact, DMDS has been registered and commercialized as a soil fumigant under the trade name Paladin^®^ EC, manufactured by Arkema Inc., and is approved for use by the U.S. Environmental Protection Agency [189,190]. Its formulation provides a practical, field-deployable alternative to methyl bromide, offering strong biocidal activity while breaking down rapidly into non-toxic sulfur compounds.

While promising, the DMDS application has considerations. Its strong odor may restrict use in some settings, and inhalation at high concentrations may pose risks to humans. Therefore, formulation and application strategies must be optimized to balance efficacy and safety.

### 7.2. Dimethyl Sulfide (DMS)

Dimethyl sulfide (DMS) is characterized by its distinctive odor, often associated with cooked cabbage or marine environments. It plays a significant role in the global sulfur cycle and has implications for climate regulation due to its involvement in cloud formation [176,191]. Certain *Burkholderia* species have been identified as producers of DMS through the catabolism of dimethylsulfoniopropionate, a compatible solute synthesized by various marine algae and some angiosperms [191,192].

The production of DMS by *Burkholderia* species holds important ecological significance across various environments. In the atmosphere, DMS plays a role in climate regulation by oxidizing into sulfate aerosols that contribute to cloud condensation nuclei, thereby influencing cloud formation and potentially affecting global temperature and precipitation patterns [176,192]. In terrestrial and plant-associated ecosystems, DMS produced by root-associated *Burkholderia* may participate in plant-microbe interactions, potentially serving as a chemical signal or modulator of microbial community dynamics. The presence and expression of volatile sulfur compound biosynthesis genes can even help distinguish species such as *B. pseudomallei* from closely related non-pathogenic species [181].

### 7.3. S-Methyl Thioacetate (SMT)

S-methyl thioacetate (SMT) is characterized by its simple thioester structure and is known for a distinctive sulfurous odor reminiscent of cabbage or garlic. *B. pyrrocinia* CNUC9, isolated from the maize rhizosphere, emits a spectrum of VOCs, including SMT, DMDS, and 2-undecanone. These emissions have been linked to enhanced germination and survival rates of *Arabidopsis thaliana* under salt stress conditions. Furthermore, exposure to the emission has been shown to alter root architecture and increase leaf area in *A. thaliana* [185]. These findings suggest a multifaceted function for SMT, with contributions to plant resilience and growth promotion under abiotic stress.

Production of SMT by *B. gladioli* was also reported. In terms of biocontrol, SMT exhibits significant fumigant toxicity against nematodes *Caenorhabditis elegans* and *M. incognita*. Specifically, the lethal concentration (LC_50_) was determined to be 1.43 μg/cm^3^ of air for *C. elegans*. In parallel comparison, SMT showed stronger fumigant toxicity against *M. incognita* than DMDS [183]. The utilization of SMT-producing bacteria, such as *Bacillus aryabhattai* [193], or the direct application of synthesized SMT could provide an eco-friendly alternative to conventional nematicides [183].

### 7.4. 2,5-Dimethylfuran

2,5-Dimethylfuran, characterized by its aromatic odor, occurs naturally in a variety of environmental contexts. In the context of *Burkholderia* species, 2,5-dimethylfuran has been identified as a significant VOC when *B. gladioli* strain BBB-01 is cultivated in potato extract-glucose medium [186]. 

2,5-Dimethylfuran exhibits inhibitory activity against several phytopathogenic fungi, including *Magnaporthe oryzae*, *Gibberella fujikuroi*, *Sarocladium oryzae*, *Phellinus noxius*, and *Colletotrichum fructicola*, as well as the human pathogen *Candida albicans* [186]. This broad-spectrum antifungal activity underscores its potential role in biological control and plant protection strategies.

### 7.5. 2-Undecanone

2-Undecanone is a medium-chain methyl ketone detected in the headspace of *B. pyrrocinia* CNUC9. Functionally, pure 2-undecanone showed dose-dependent effects on plants: low doses promoted *Arabidopsis thaliana* root and shoot growth, while high doses inhibited growth—demonstrating that even a minor VOC can have bioactivity [185]. Additionally, 2-undecanone has been identified as an antifungal VOC produced by *Burkholderia*. In *B. ambifaria*, the compound reduced the growth of *Rhizoctonia solani* and *Alternaria alternata* at higher doses [182].

### 7.6. Methyl Salicylate

Methyl salicylate is a VOC known for its characteristic wintergreen aroma. While it is predominantly produced by various plant species, certain bacterial species, including members of the *Burkholderia* genus, have also been identified to synthesize this compound.

Research indicates that *B. cenocepacia* strain ETR-B22 emits methyl salicylate among many aromatic VOCs, including methyl anthranilate, methyl benzoate, benzyl propionate, benzyl acetate, 3,5-di-tert-butylphenol, allyl benzyl ether, and benzyl benzoate, all of which have demonstrated inhibitory effects against several fungal plant pathogens [178,187].

In plants, methyl salicylate serves as a signaling molecule involved in systemic acquired resistance, a key defense mechanism against pathogens. It also plays a role in inter-plant communication, alerting neighboring plants to potential threats [194,195]. The production of methyl salicylate by *Burkholderia* species highlights their potential as biological control agents in agriculture. By inhibiting fungal pathogens, these bacteria could reduce the reliance on chemical fungicides, promoting more sustainable farming practices.

## 8. Discussion and Perspectives

The genus *Burkholderia* stands at a critical crossroads of risk and opportunity. While many species are recognized for their roles as plant and human pathogens, recent advances in genomics, synthetic biology, and biotechnology are rapidly transforming our approach to these bacteria. Increasingly, researchers are looking beyond the risks to explore *Burkholderia* as a treasure trove of biotechnologically valuable metabolites and ecological functions. Many environmental and plant-associated species act as plant growth promoters, nutrient mobilizers, and potent biocontrol agents. In fact, the phylogeny illustrated in Figure 1 reveals a clear clade-level asymmetry: the *Burkholderia* sensu stricto clade concentrates species with both antibacterial and antifungal activities, and some of these occur across multiple habitats, including soil, water, plants, and animals. This ecological profile is typical of metabolite-rich generalists. By contrast, *Paraburkholderia* skews toward plant-associated lifestyles and a sparser secondary metabolite footprint. Species spanning multiple habitats and showing dual antibacterial and antifungal phenotypes should be prioritized as “secondary metabolite generalists,” because ecological breadth and dual activity correlate with metabolite richness and competitive fitness. From a metabolite-discovery perspective, this pattern suggests that *Burkholderia* sensu stricto should be prioritized as primary sources of potent chemistry under appropriate containment, while plant-beneficial *Paraburkholderia* and related environmental lineages provide complementary, lower-risk reservoirs of bioactive traits for agricultural applications.

Compared with classic natural-product workhorses such as *Streptomyces* and *Bacillus*, *Burkholderia* occupies a distinctive but more constrained niche in practical applications. *Streptomyces* spp. have historically dominated antibiotic discovery and are generally regarded as low-risk soil saprophytes, while many *Bacillus* spp. combine metabolite production with spore-based formulations that are straightforward to stabilize and register as biopesticides. By contrast, *Burkholderia* sensu stricto species have large, multireplicon genomes with rich, often cryptic BGC repertoires and frequently produce structurally unusual metabolites with potent antibacterial or antifungal activity [3,4,37,128]. This combination of genomic complexity and chemical diversity makes them particularly attractive for the discovery of new antimicrobial scaffolds. At the same time, some members of this group are opportunistic or frank pathogens with intrinsic multidrug resistance, so *Burkholderia*-based biotechnology must balance metabolic novelty and ecological competitiveness against heightened regulatory scrutiny, careful strain selection (e.g., favoring *Paraburkholderia* and other strictly environmental lineages for field applications), and the need to attenuate or avoid virulence- and resistance-associated traits.

The capacity of these bacteria to colonize the rhizosphere and compete with harmful microbes makes them attractive candidates for next-generation biocontrol products. For example, cepacin-producing *B. ambifaria* strains suppress pea damping-off caused by *Globisporangium ultimum* [95,137], DMDS-producing *B. ambifaria* H8 can control maize stalk rot [175], illustrating how *Burkholderia*-derived antibiotics and VOCs can function as seed or soil treatments within integrated disease management strategies. However, deploying *Burkholderia* in biotechnology also carries non-trivial biosafety concerns: members of the Bcc are opportunistic, often multidrug-resistant, pathogens of people with CF and other underlying conditions, sometimes associated with poor clinical outcomes, while *B. pseudomallei* and *B. mallei* cause severe, difficult-to-treat infections that require prolonged antimicrobial therapy [3,5,7,20,43]. Their high intrinsic resistance, diverse virulence factors, and broad ecological range demand rigorous risk assessment, regulatory compliance, and long-term biosafety testing for any translational application [196,197]. To achieve this, removing or attenuating pathogenicity determinants could be crucial, for example, by deleting secretion systems or toxin/virulence metabolite pathways. In *Burkholderia* models, such targeted edits consistently reduce virulence, including T3SS *bscN* mutants in *B. cepacia* [198], loss of the T6SS effector TecA in *B. cenocepacia* [199], toxoflavin-deficient *B. glumae* [53], and disruption of the malleilactone cluster in the *B. pseudomallei* group [116,117]. In practical terms, this means that translational applications should rely on carefully characterized, low-risk species and strains, and should be designed to minimize environmental and clinical exposure, especially in contexts involving vulnerable patient groups or the food chain.

Unlocking the full metabolic potential of *Burkholderia* requires activation of cryptic or silent BGCs [200], which often remain unexpressed under standard laboratory conditions. Advanced genome editing and recombineering technologies, such as targeted mutagenesis (host-based homologous recombination, phage-based recombineering, CRISPR-Cas systems), untargeted mutagenesis (transposon-based approaches), and heterologous expression in surrogate hosts (including *Burkholderia* chassis), provide practical means to implement both attenuation and activation strategies without compromising desired traits [201,202,203,204]. For example, LTTRs play a pivotal role in modulating the expression of these silent clusters [140]. By engineering LTTRs or manipulating their regulatory networks, researchers have succeeded in activating previously inaccessible BGCs, leading to the discovery of novel antibiotics and other bioactive metabolites. Combining LTTR modulation with genome mining, promoter engineering, and advanced omics approaches offers a promising strategy for expanding *Burkholderia’s* arsenal of valuable natural products for agricultural and pharmaceutical use. Given the increasing availability of complete genome sequences, functional genomics tools, and efficient transformation protocols, *Burkholderia* is now highly amenable to such integrated activation and attenuation strategies.

Looking forward, the integration of high-throughput genome mining, synthetic biology, and systems biology approaches will further accelerate the discovery and optimization of *Burkholderia*-derived bioactive compounds for the agriculture and medicine sectors. Metabolic engineering could also be used to boost the yields of key antifungal metabolites or VOCs. For instance, the antifungal glycopeptide occidiofungin from *B. contaminans* has shown potent, broad-spectrum activity against human and plant-pathogenic fungi and is being investigated for both clinical and crop protection applications [104,145,146], while biosynthetic and media engineering of *Burkholderia* spp. has enabled improved production of spliceostatin/thailanstatin-type pre-mRNA splicing inhibitors as anticancer leads [118,164,165]. In bioindustrial and environmental contexts, rhamnolipid biosurfactants from *Burkholderia* spp. are being explored for green formulations and bioremediation of persistent pollutants [124,173], while aromatic-degrading *Paraburkholderia* strains such as *P. xenovorans* LB400 are used for the breakdown of persistent organic pollutants [37]. Beneficial species are already being tested as biofertilizers, nitrogen fixers, and bioremediators, but coordinated efforts linking molecular insights with field-level trials and risk mitigation will be essential to realize these applications responsibly. In summary, the future of *Burkholderia* research lies not only in managing its risks but also in unlocking its vast biotechnological promise. The rational design of safe, effective, and application-tailored strains, coupled with stringent biosafety frameworks, could position *Burkholderia* as a cornerstone of next-generation sustainable agriculture, biotechnology, and medicine.

## Figures and Tables

**Table 1:** Overview of secondary metabolites produced by *Burkholderia* species, summarizing their biosynthetic system, mode of action, prospective applications, corresponding producing strains, and references.

**Cepaciachelin (NRPS)**		
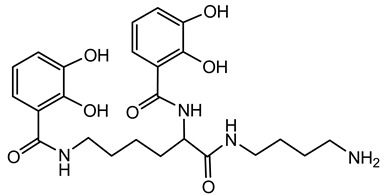	Mode of action	Iron chelation/sequestration
	Potential applications	Biotech chelation
	Producing strains	*Burkholderia ambifaria* AMMD, *B. cepacia* PHP7
	Core biosynthetic genes/enzymes	2,3-dihydroxy benzoic acid biosynthesis enzymes, NRPSs
	References	[70,71]
**Ornibactin (NRPS)**		
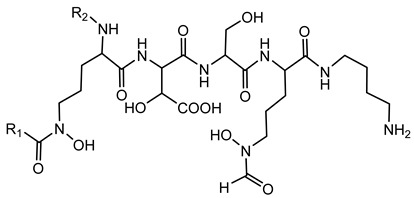 Ornibactin C4 (R1=CH_2_CH(OH)CH_3_; R2=H) Ornibactin C6 (R1=CH_2_CH(OH)(CH_2_)_2_CH_3_; R2=H) Ornibactin C8 (R1=CH_2_CH(OH)(CH_2_)_4_CH_3_; R2=H) Malleobactin E (R1=H; R2=CHO)	Mode of action	Iron chelation/sequestration
	Potential applications	Therapeutic potential, bioimaging potential
	Producing strains	*Burkholderia contaminans* MS14, *B. cenocepacia* K56-2
	Core biosynthetic genes/enzymes	*orb* cluster-*OrbI*, *OrbJ*; *pvdA*: encoding _L-_ornithine N5-oxygenase
	References	[41,42,72,73]
**Malleobactin (NRPS)**		
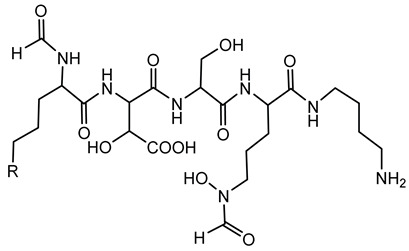 Malleobactin A (R=NO_2_) Malleobactin B (R=NHOH) Malleobactin C (R=NO)	Mode of action	Iron chelation/sequestration
	Potential applications	Anti-virulence target
	Producing strains	*B. pseudomallei* K96243
	Core biosynthetic genes/enzymes	*mba* cluster-*mbaA*: NRPS, *mbaC*: ornithine monooxygenase, *mbaE*: formyltransferase; *mbaH*: hydroxylase
	References	[74,75,76]
**Bolagladin (NRPS)**		
Bolagladin A (R=CH_3_) Bolagladin B (R=CH_2_CH_3_) 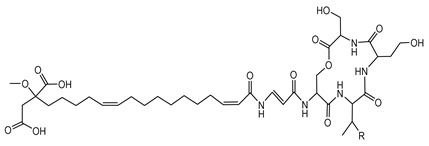	Mode of action	Iron chelation/sequestration
	Potential applications	Agricultural potential, pharmaceutical potential
	Producing strains	*B. gladioli* BCC0238, *B. gladioli* BCC1622
	Core biosynthetic genes/enzymes	*bol* cluster (*bolA-T*)*-bolH*: tetramodular NRPS, *bolR*: citrate synthase-like enzyme
	References	[77]
**Gramibactin (NRPS)**		
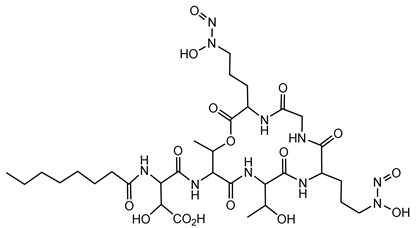	Mode of action	Iron chelation/sequestration
	Potential applications	Biofertilizer for providing nitric oxide
	Producing strains	*Paraburkholderia graminis* C4D1M
	Core biosynthetic genes/enzymes	*grb* cluster-*grbD* and *grbE*: graminine biosynthesis
	References	[78,79,80]
**Gladiolin (*trans*-AT PKS)**		
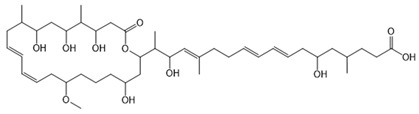	Mode of action	Bacterial RNA polymerase inhibition
	Potential applications	Antibacterial therapy
	Producing strains	*B. gladioli* BCC0238
	Core biosynthetic genes/enzymes	*gbnD1-D6* cluster-*gbnD1*: modular PKS
	References	[81,82]
**Enacyloxin IIa (Hybrid *cis*-AT/*trans*-AT PKS)**		
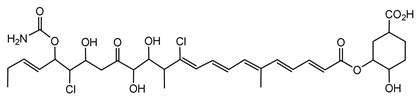	Mode of action	Inhibition of elongation factor Tu
	Potential applications	Antibacterial therapy
	Core biosynthetic genes/enzymes	enacyloxin cluster-Bamb_5919-5925: PKS, Bamb_5932: enacyloxin oxidase
	Producing strains	*B. ambifaria* AMMD, *B. gladioli* BCC1701
	References	[82,83,84]
**Gladiostatin/Gladiofungin (*trans*-AT PKS)**		
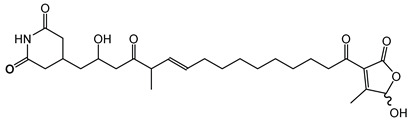	Mode of action	Unknown
	Potential applications	Antifungal, anticancer potential
	Producing strains	*B. gladioli* BCC0238, *B. gladioli* BCC1622
	Core biosynthetic genes/enzymes	*gds* (or *gla*) cluster-*gdsE* and *gdsF* (or *glaD*): PKS, *glaG*: enoyl reductase
	References	[85,86]
**Icosalide A (NRPS)**		
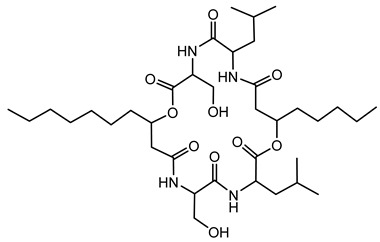	Mode of action	Unknown
	Potential applications	Agricultural potential
	Producing strains	*B. gladioli* BCC0238, *B. gladioli* HKI0739
	Core biosynthetic genes/enzymes	*icoA* or *icoS*: NRPSs
	References	[87,88]
**Burkholdines (NRPS)**		
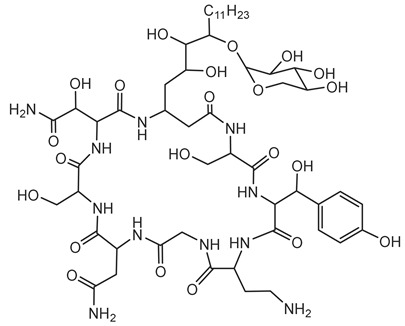	Mode of action	Unknown (putative β-glucan synthase inhibition)
	Potential applications	Antifungal
	Producing strains	*B. ambifaria* 2.2N
	Core biosynthetic genes/enzymes	Unknown
	References	[89,90]
**Pyrrolnitrin (Shikimate pathway derivative)**		
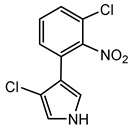	Mode of action	Fungal electron transport system inhibition
	Potential applications	Agricultural potential, therapeutic applications
	Producing strains	*B. cepacia* NB-1, *B. cepacia* JBK9, *B. contaminans* NZ
	Core biosynthetic genes/enzymes	*prnABCD* operon-*prnD*: aminopyrrolnitrin oxidase
	References	[91,92,93,94]
**Cepacin (Polyyne)**		
Cepacin A 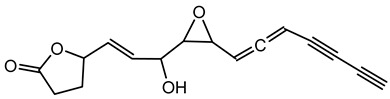 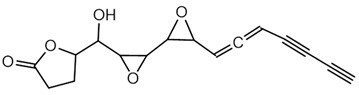 Cepacin B	Mode of action	Fungal cell wall disruption
	Potential applications	Antifungal, anti-oomycete
	Producing strains	*B. ambifaria* BCC0191
	Core biosynthetic genes/enzymes	*ccnA-P* cluster-*ccnJ*: fatty acyl-AMP ligase
	References	[95]
**Thailandenes (PKS)**		
Thailandene A 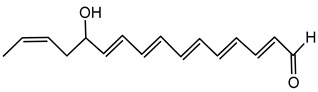 Thailandene B 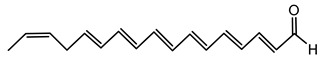 Thailandene C 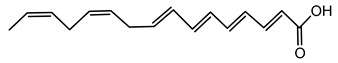	Mode of action	Membrane disruption
	Potential applications	Antibacterial, anti-yeast
	Producing strains	*Burkholderia thailandensis*
	Core biosynthetic genes/enzymes	*orgA-M* cluster-*orgA-C*: PKS
	References	[96]
**Thailandamide (Hybrid PKS–NRPS)**		
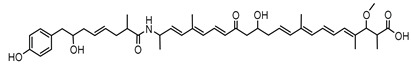	Mode of action	Fatty-acid synthesis inhibition
	Potential applications	Antimicrobial applications
	Producing strains	*B. thailandensis* E264
	Core biosynthetic genes/enzymes	*thaA-R* cluster-*thaG-Q*: PKS
	References	[97,98,99]
**Bactobolin (Hybrid PKS–NRPS)**		
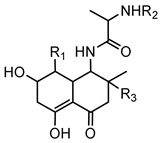 A: R_1_=OH; R_2_=H; R_3_=CHCl_2_ B: R_1_=OH; R_2_=Ala; R_3_=CHCl_2_ C: R_1_=H; R_2_=H; R_3_=CHCl_2_ D: R_1_=H; R_2_=Ala; R_3_=CHCl_2_ E:R_1_=OH;R_2_=Ala-Ala; R_3_=CHCl_2_ F: R_1_=H; R_2_=Ala-Ala; R_3_=CHCl_2_ G: R_1_=H; R_2_=H; R_3_=CH_2_Cl H: R_1_=H; R_2_=Ala; R_3_=CH_2_Cl	Mode of action	Protein synthesis inhibition
	Potential applications	Antimicrobial applications
	Producing strains	*B. thailandensis* E264
	Core biosynthetic genes/enzymes	*btaA-U* cluster-*btaK*: NRPS
	References	[100,101]
**4-Hydroxy-3-methyl-2-alkylquinolines (HMAQs) (Alkylquinolone pathway)**		
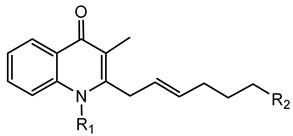 HMAQ 1: R1=H; R2=CH_3_ HMAQ 2: R1=H; R2=CH_2_CH_3_ HMAQ 3: R1=H; R2=CH_2_CH_2_CH_3_	Mode of action	Unknown (Putative: proton motive force dissipation)
	Potential applications	Antimicrobial applications
	Producing strains	*B. thailandensis*, *B. pseudomallei*, *B. ambifaria*
	Core biosynthetic genes/enzymes	*hmqABCDEFG* operon-*hmqA*: anthranilate–CoA ligase, *hmqG*: methyltransferase
	References	[102,103]
**Occidiofungin (NRPS)**		
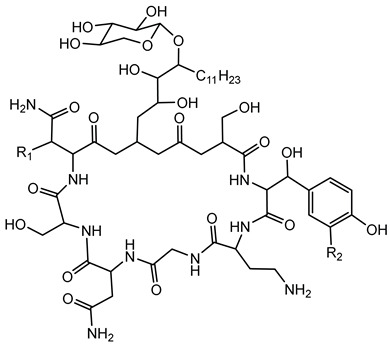 A: R_1_=H; R_2_=H B: R_1_=OH; R_2_=H C: R_1_=H; R_2_=Cl D: R_1_=OH; R_2_=Cl	Mode of action	Disruption of actin-mediated functions
	Potential applications	Antifungal, potential therapeutic agent
	Producing strains	*B. contaminans* MS14, *Burkholderia pyrrocinia* Lyc2
	Core biosynthetic genes/enzymes	*ocfA–J* cluster-*ocfC*: xylosyltransferase
	References	[104,105,106,107,108]
**Bongkrekic acid (PKS)**		
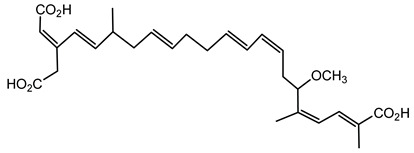	Mode of action	Mitochondrial toxin
	Potential applications	Food safety concerns
	Producing strains	*B. gladioli* pv. *cocovenenans*
	Core biosynthetic genes/enzymes	*bonA-M* cluster-*bonL*: cytochrome P450 monooxygenase
	References	[109,110]
**Toxoflavin (GTP-derived azapteridine pathway)**		
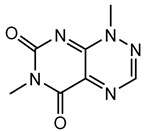	Mode of action	Disruption of cellular redox homeostasis
	Potential applications	Agricultural applications
	Producing strains	*B. glumae* BGR1, *B. gladioli* HDXY-02
	Core biosynthetic genes/enzymes	*toxABCDE* operon-*toxA*: methyltransferase
	References	[111,112,113,114,115]
**Malleilactone (Hybrid PKS–NRPS)**		
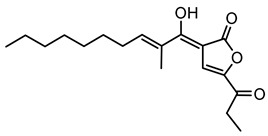	Mode of action	Unknown
	Potential applications	Anti-virulence therapy
	Producing strains	*B. thailandensis*, *B. pseudomallei*, *B. mallei*
	Core biosynthetic genes/enzymes	*mal* cluster-*malA* and *malF*: PKSs,
	References	[116,117]
**Thailanstatin (Hybrid PKS–NRPS)**		
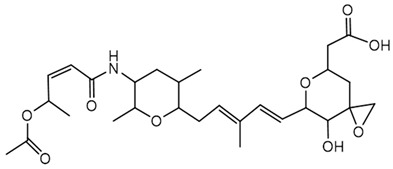	Mode of action	Spliceosome inhibition
	Potential applications	Anticancer potential
	Producing strains	*B. thailandensis* MSMB43
	Core biosynthetic genes/enzymes	*tstA-R* cluster-*tstC*: PKS, *tstP*: dioxygenase
	References	[118,119]
**Rhizoxin (Hybrid PKS–NRPS)**		
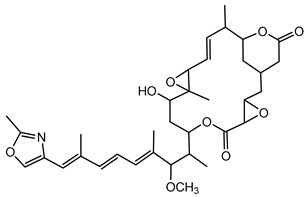	Mode of action	Mitosis inhibition
	Potential applications	Anticancer potential
	Producing strains	*Burkholderia rhizoxinica* HKI 0454
	Core biosynthetic genes/enzymes	*rhi* cluster-PKSs and NRPSs
	References	[120,121,122,123]
**Rhamnolipids** (**Glycolipid pathway)**		
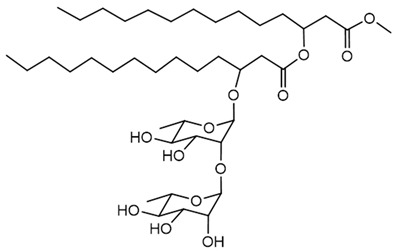	Mode of action	Membrane disruption
	Potential applications	Antimicrobial applications, biosurfactant in green chemistry
	Producing strains	*B. thailandensis* E264, *B. glumae* AU6208
	Core biosynthetic genes/enzymes	*rhl* cluster-*rhlA*: 3-(3-hydroxyalkanoyloxy) alkanoic acid synthase, *rhlB* and *rhlC*: rhamnosyltransferase
	References	[124,125,126,127]

**Table 2:** VOCs emitted by various *Burkholderia* species. The table summarizes their chemical structure, biological activities, and associated references.

VOCs	Chemical Structure	Activity or Function	Producers	References
Dimethyl disulfide	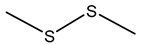	Antifungal, nematicidal, plant growth promotion	*B. ambifaria* H8, *B. gladioli* BBB-01, *B. pyrrocinia* JK-SH007	[175,176,177]
Dimethyl trisulfide	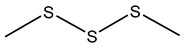	Antifungal	*Burkholderia vietnamiensis* B418 *B. cenocepacia* ETR-B22, *Burkholderia* sp. AD24	[178,179,180]
Dimethyl sulfide	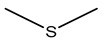	Species-specific volatile marker	*B. pseudomallei*, *B. thailandensis*	[181,182]
S-Methyl thioacetate	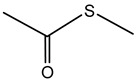	Antifungal, antibacterial, nematicidal	*B. gladioli*, *B. pyrrocinia* CNUC9	[183,184,185]
Benzothiazole	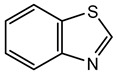	-	*B. pyrrocinia* JK-SH007	[177]
Pyrazines	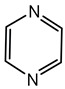	Antifungal, signaling, and virulence regulation	*Burkholderia* sp. AD24	[180]
2-Undecanone	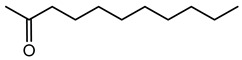	Antifungal, plant growth promotion	*B. ambifaria*	[182]
2-Nonanone	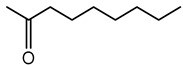	Antifungal	*B. ambifaria*	[182]
2-Aminoacetophenone	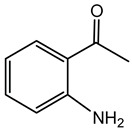	Antibiotic resistance modulation	*B. ambifaria*	[182]
2,5-Dimethylfuran	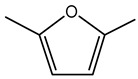	Antifungal	*B. gladioli* BBB-01	[186]
Methyl salicylate	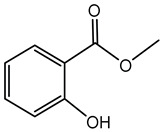	Antifungal	*B. cenocepacia* ETR-B22	[178]

## Data Availability

No new data were created or analyzed in this study. Data sharing is not applicable to this article.
